# Anemia in tuberculosis cases: A biomarker of severity?

**DOI:** 10.1371/journal.pone.0245458

**Published:** 2021-02-02

**Authors:** Edson Beyker de Mendonça, Carolina AranaStanis Schmaltz, Flavia Marinho Sant’Anna, Alexandre Gomes Vizzoni, Daniela Palheiro Mendes-de-Almeida, Raquel de Vasconcellos Carvalhaes de Oliveira, Valeria Cavalcanti Rolla

**Affiliations:** 1 Hematology Section, Clinical Analysis Laboratory, Evandro Chagas National Institute of Infectious Diseases, Fiocruz, Rio de Janeiro/RJ, Brazil; 2 Clinical Research Laboratory on Mycobacteria, Evandro Chagas National Institute of Infectious Diseases, Fiocruz, Rio de Janeiro/RJ, Brazil; 3 Hemotherapy Section, Evandro Chagas National Institute of Infectious Diseases, Fiocruz, Rio de Janeiro/RJ, Brazil; 4 Clinical Epidemiology Laboratory, Evandro Chagas National Institute of Infectious Diseases, Fiocruz, Rio de Janeiro/RJ, Brazil; The University of Georgia, UNITED STATES

## Abstract

**Introduction:**

Anemia is a common condition at tuberculosis diagnosis, and there is evidence that its prevalence is higher in patients with tuberculosis than in those infected with *Mycobacterium tuberculosis* and healthy controls. Information about anemia during tuberculosis diagnosis is still scarce in the Brazilian population. The aim of this study was to describe the prevalence of anemia in patients with tuberculosis cared for at a referral center and its association with clinical forms of tuberculosis and other characteristics of these patients.

**Materials and methods:**

This was a retrospective cross-sectional study of tuberculosis patients diagnosed from January 2015 to December 2018 at the Clinical Research Laboratory on Mycobacteria (LAPCLIN-TB) of Evandro Chagas National Institute of Infectious Diseases (INI)/Oswaldo Cruz Foundation (Fiocruz). A database of an ongoing cohort study underway at this service since 2000 provided the baseline information on tuberculosis cases extracted from a visit template. Exploratory and logistic regression analyses were performed to verify associations between anemia and demographic characteristics, socioeconomic status, clinical conditions, and laboratory results.

**Results:**

Of the 328 cases reviewed, 70 were excluded, with258 retained. The prevalence of anemia was 61.2% (27.5% mild, 27.5% moderate and 6.2% severe). Among patients with anemia, 60.8% had normochromic normocytic anemia, and 27.8% showed hypochromic microcytic anemia. In logistic regression analysis, anemia was associated with a history of weight loss >10%, hospitalizations, coinfection with HIV, increased platelet count and microcytosis. Anemia was more frequent in the most severe clinical forms, such as meningeal and disseminated tuberculosis.

**Conclusions:**

Anemia was highly prevalent in tuberculosis patients at diagnosis, predominantly as normochromic normocytic anemia and in mild and moderate forms. It was associated with baseline characteristics and conditions indicative of severe disease, suggesting that anemia could be a biomarker of tuberculosis severity.

## Introduction

Approximately 10 million people worldwide developed tuberculosis (TB)in 2019. TB is one of the top ten causes of death and the leading cause of death by a single infectious agent. Brazil is among the 20 countries with the highest absolute numbers of TB cases. In 2019, the estimated TB incidence was 96,000 cases, with an upward trend from 2016 to 2019 [1]. Rio de Janeiro is the state with the second highest burden of TB in the country, having had 66.3 cases/100,000 habitants in 2018, and the Rio de Janeiro municipality has an incidence rate of 89.9/100,000 habitants [2]. Anemia affects approximately a quarter of the world's population, impairing patients’ health and compromising social and economic development. Multifactorial causes may contribute, either individually or in groups, to anemia. Among these causes are infectious diseases [3].

Anemia is a common condition at TB diagnosis, with prevalence estimates ranging from 9.5% to 96%, depending on the location, study design and population studied [4]. There is evidence that anemia is more frequent in TB patients than in TB contacts infected with *Mycobacterium tuberculosis* [5] and healthy controls [6, 7]. Hemoglobin (Hb) levels tend to decrease as acid-fast bacilli (AFB) smear positivity increases [8], and the presence of anemia at TB diagnosis has been associated with delayed smear negativity [6, 9]. Anemia has been associated with unfavorable TB outcomes [10] and a higher risk of lethality among TB patients [11, 12], and its prevalence is significantly higher in HIV-TB than in HIV-negative TB patients [9, 11, 13–16], suggesting an association of anemia with severe cases of TB.

In Brazil, despite the high prevalence of TB in the population, there are few studies evaluating anemia and the factors associated with its occurrence. The two Brazilian studies that addressed anemia were performed in hospitalized pulmonary TB patients [17, 18] with poor health status. In contrast, patients with less severe TB illness have not yet been studied in Brazil.

The aim of this study was to describe the prevalence of anemia at TB diagnosis in a referral service for TB in Rio de Janeiro, classifying anemia according to its severity and morphological characteristics, and to identify its associated factors.

## Methods

### Population and study design

A retrospective cross-sectional study was carried out between January 2015 and December 2018 at the Clinical Research Laboratory on Mycobacteria (LAPCLIN-TB) of Evandro Chagas National Institute of Infectious Diseases (INI)/Oswaldo Cruz Foundation (Fiocruz), in Rio de Janeiro, Brazil. It is an infectious disease hospital located in a central area of Rio de Janeiro that treats many infectious diseases, including TB.

LAPCLIN-TB has maintained an open cohort of TB patients diagnosed and treated since 2000. There is a well-defined healthcare protocol for TB, including interviews and systematic collection of demographic, socioeconomic, clinical and laboratory data.

A TB dataset from this previous cohort study (assessment of survival-associated factors in TB individuals, HIV-infected or noninfected) was revised, and patients were selected according to the following criteria: the inclusion criteria were 18 years or older and a TB diagnosis based on the criteria described (see definition in the next section), and the exclusion criteria were (a) cutaneous, renal, bone, ocular, mammary or other less common forms of TB; (b) missing demographic, socioeconomic or clinical data; (c) complete blood count not available from 10 days before to 10 days after starting TB treatment; and (d) history of blood transfusion in the last 4 months.

### Definitions

Anemia was defined according to the World Health Organization (WHO) guideline criteria: Hb value <13 g/dL for men and <12 g/dL for women. The degrees of anemia were classified according to the WHO criteria: mild anemia: 11.0–12.9 g/dL (men) and 11.0–11.9 g/dL (women), moderate anemia: 8.0–10.9 g/dL (both sexes), and severe anemia: <8.0 g/dL (both sexes) [19].

The mean corpuscular volume (MCV) value was used to classify anemia as microcytic (MCV<80 fL), normocytic (MCV between 80 and 100 fL) and macrocytic (MCV>100 fL). The mean corpuscular hemoglobin (MCH) value was used to classify anemia as hypochromic (if MHC <26 pg) or normochromic (if MCH ≥26 pg) [20].

Patients were considered to have TB if there was a positive culture for *Mycobacterium tuberculosis*, a positive Xpert MTB-RIF, or a positive therapeutic test (improvement of clinical signs and symptoms of TB under specific TB treatment, with exclusion of other diseases and with a positive or negative smear).

Clinical presentation of TB was classified as pleuropulmonary (when restricted to the lungs and/or pleura), extrapulmonary (when only one extrapulmonary site was affected) or disseminated (when it involved at least 2 noncontiguous sites).

Along with the severity of TB, clinical presentation was also classified according to the infection site: (a) ganglionic; (b) laryngeal + pulmonary + pleural and (c) disseminated + meningeal (included in the same group due to severity).

Comorbidities were recorded as one or more of these chronic diseases: systemic arterial hypertension, diabetes mellitus, digestive disease, degenerative disease, hepatitis, kidney disease, neurological disease, chronic obstructive pulmonary disease and neoplasia/cancer.

CD4 cell counts were dichotomized into<200 and ≥200 cells/μL based on the Centers for Disease Control and Prevention (CDC) and Brazilian Ministry of Health guidelines for the definition of AIDS to express advanced immunodeficiency among HIV-infected cases [21].

### Data sources/measurements

The data collected included sex, age, race, family income (one Brazilian minimum wage [MW] was $270/month, the average value in the period in which patients were included), schooling, area of residence, alcohol abuse, tobacco use history, drug abuse, height and weight (used to calculate body mass index [BMI]), Karnofsky score, history of weight loss, comorbidities, HIV infection, antiretroviral therapy (ART), opportunistic diseases, hospitalizations (current and recent [≤24 months] were considered), surgeries, previous TB treatment, clinical form of TB and the results of laboratory tests (complete blood count; smears using the Ziehl-Neelsen method, Xpert MTB/RIF® assay and culture [Lowenstein–Jensen medium or MGIT]; C-reactive protein and albumin; HIV testing; CD4 cell count; and viral load).

### Statistical analysis

The frequencies, medians and interquartile ranges (IQRs) of categorical and continuous variables are described. In bivariate analysis, associations of categorical variables with anemia were calculated by Pearson's chi-square test or Fisher's exact test (if the expected count was<5 in the cell of a 2x2 table). Due to the rejection of the normality assumption by the Shapiro-Wilk test, the Mann-Whitney test was employed to test for associations of continuous variables with anemia. We used a binary logistic regression model (logit model) to estimate the effects of variables according to the outcome “anemia” (presence or absence). The inclusion criteria for the multicovariate model (with several explanatory variables) were p-values <0.20 in the single covariate model (with one explanatory variable). Before modeling, we excluded variables/categories with a 2x2 table cell count <5 in the bivariate analysis (disseminated/meningeal clinical form), more than 10% missing values (income, BMI, ART status and CD4 cell count) or a confounder effect (“Morphological classification of red blood cells based on Hb content”).

We provide the effects of the single and multicovariate models (crude and adjusted odds ratios [OR], respectively). The Hosmer and Lemeshow test did not show a lack of fit. Due to critical use of the p-value, we added 95% confidence intervals (CIs) to the estimates [22].

Statistical analyses were performed using Statistical Package for Social Sciences (SPSS) version 16.0.

### Ethics

This retrospective cross-sectional study was approved by the Research Ethics Committee of INI/Fiocruz (number of approval: 96128318.6.0000.5262). All participating patients signed a written informed consent form when they agreed to participate in the TB survival prospective cohort study from which data were collected for this study (designated "Assessment of survival-associated factors in TB individuals, HIV-infected or non-infected” and approved by the Research Ethics Committee of INI under the number 88604818.6.0000.5262), and the study was conducted according to the principles expressed in the Declaration of Helsinki.

## Results

### General characteristics of the study population

Three hundred twenty-eight participants were reviewed, and 70 were excluded. The reasons for exclusion were as follows: less common extrapulmonary TB forms, such as cutaneous (15), ocular (7), breast (9), epididymal (2), bone (3), and renal (1); blood transfusions in the last four months (2); Hb results not available from 10 days before to 10 days after starting TB treatment (18); and missing demographic, socioeconomic, or clinical data (13). The final sample included 258 patients.

The median age was 36.5 years (IQR 28.0–50.0), with a predominance of men (63.6%; 95% CI = 57.4–69.4), nonwhite subjects (54.1%; 95% CI = 47.8–60.3), subjects living in the Rio de Janeiro municipality (66.7%; 95% CI = 60.5–72.4) and outside the slum area (69.0%; 95% CI = 62.1–75.4), with elementary schooling or less (60.0%; 95% CI = 53.8–66.1) and with a family income up to 2 Brazilian minimal wages (MW) (66.0%; 95% CI = 58.8–72.6). The predominant clinical form was pleuropulmonary (72.9%; 95% CI = 67.0–78,2), and most cases were confirmed with culture and/or Xpert MTB-RIF (77.5%; 95% CI = 71.9–82.5). HIV serology was positive in 32.6% (95% CI = 26.9–38.6) of cases, and 26.8% (95% CI = 21.4–32.7) were hospitalized on the day of screening or had a recent hospitalization (within the last 2 years).

### Prevalence of anemia and baseline characteristics

The prevalence of anemia was 61.2% (95% CI = 55.0–67.2); 27.5% (95% CI = 22.2–33.4) of the subjects had mild anemia, 27.5% (95% CI = 22.2–33.4) moderate anemia and 6.2% (95% CI = 3.6–9.9) severe anemia. The median Hb level was 12.1 g/dL (IQR 10.3–13.4), with the lowest value of 5.8 g/dL and the highest value of 16.5 g/dL. Table 1 shows the status of anemia in patients at TB therapy initiation according to their baseline characteristics.

**Table 1:** Demographic characteristics, socioeconomic status, clinical condition and laboratory results for participants with and without anemia.

	No anemia		Anemia		p-value^A^
	n	% (95% CI)	n	% (95% CI)	
**Age, years**					**0.581**
18–35	50	50.0 (39.8–60.2)	69	43.7 (35.8–51.8)	
36–49	25	25.0 (16.9–34.7)	47	29.7 (22.7–37.5)	
>49	25	25.0 (16.9–34.7)	42	26.6 (19.9–34.2)	
**Sex**					**0.895**
Female	37	37.0 (27.6–47.2)	57	36.1 (28.6–44.1)	
Male	63	63.0 (52.8–72.4)	101	63.9 (55.9–71.4)	
**Race**					**0.002**
White	58	58.0 (47.7–67.8)	60	38.2 (30.6–46.3)	
Nonwhite	42	42.0 (32.2–52.3)	97	61.8 (53.7–69.4)	
**Schooling**					**0.002**
None/incomplete elementary	19	19.0 (11.8–28.1)	50	31.6 (24.5–39.5)	
Complete elementary	28	28.0 (19.5–37.9)	58	36.7 (29.2–44.7)	
High school/university	53	53.0 (42.8–63.1)	50	31.6 (24.5–39.5)	
**Income**					**0.064**
0–2 MW^B^	44	57.9 (46.0–69.1)	84	71.2 (62.1–79.2)	
>2 MW^B^	32	42.1 (30.9–54.0)	34	28.8 (20.8–37.9)	
**Living in slum area**					**0.639**
No	57	71.2 (60.0–80.8)	79	67.5 (58.2–75.9)	
Yes	23	28.8 (19.2–40.0)	38	32.5 (24.1–41.8)	
**Tobacco use**					**0.768**
Never	51	51.5 (41.3–61.7)	82	52.9 (44.7–61.0)	
Quit	30	30.3 (21.5–40.4)	41	26.5 (19.7–34.1)	
Current	18	18.2 (11.1–27.2)	32	20.6 (14.6–27.9)	
**Alcohol**					**0.005**
Never	40	40.8 (31.0–51.2)	42	27.4 (20.6–35.2)	
Quit	25	25.5 (17.2–35.3)	70	45.8 (37.7–54.0)	
Current	33	33.7 (24.4–43.9)	41	26.8 (20.0–34.5)	
**Drug use**^**C**^					**0.024**
No	81	83.5 (74.6–90.3)	109	70.3 (62.5–77.4)	
Yes, noninjecting	16	16.5 (9.7–25.4)	46	29.7 (22.6–37.5)	
**BMI, kg/m**^**2**^					**0.014**
<18.5	10	12.3 (6.1–21.5)	33	27.0 (19.4–35.8)	
≥18.5	71	87.7 (78.5–93.9)	89	73.0 (64.2–80.6)	
**Karnofsky score**					**<0.001**
≤80	38	38.4 (28.8–48.7)	103	66.5 (58.4–73.8)	
90 or 100	61	61.6 (51.3–71.2)	52	33.5 (26.2–41.6)	
**Weight loss history (>10%)**					**<0.001**
No	82	82.0 (73.1–89.0)	71	45.2 (37.3–53.4)	
Yes	18	18.0 (11.0–26.9)	86	54.8 (46.6–62.7)	
**Comorbidities**^**D**^					**0.290**
No	66	66.7 (56.5–75.8)	93	59.2 (51.1–67.0)	
Yes	33	33.3 (24.2–43.5)	64	40.8 (33.0–48.9)	
**Recent surgery (≤24 months)**					**0.607**
No	91	91.9 (84.7–96.4)	147	94.2 (89.3–97.3)	
Yes	8	8.1 (3.6–15.3)	9	5.8 (2.7–10.7)	
**Previous TB treatment**					**0.037**
No	82	82.8 (73.9–89.7)	112	71.3 (63.6–78.3)	
Yes	17	17.2 (10.3–26.1)	45	28.7 (21.7–36.4)	
**Hospitalization**					**<0.001**
No	90	90.9 (83.4–95.8)	96	61.9 (53.8–69.6)	
Yes	9	9.1 (4.2–16.6)	59	38.1 (30.4–46.2)	
**HIV infection status**					**<0.001**
Noninfected	85	85.0 (76.5–91.4)	89	56.3 (48.2–64.2)	
Infected	15	15.0 (8.6–23.5)	69	43.7 (35.8–51.8)	
**ART status**					**<0.001**
Naive	5	33.3 (11.8–61.6)	33	50.0 (37.4–62.6)	
Interrupted	2	13.3 (1.7–40.5)	27	40.9 (29.0–53.7)	
Current	8	53.3 (26.6–78.7)	6	9.1 (3.4–18.7)	
**CD4 cell count, cells/μL**					**0.020**
<200	4	26.7 (7.8–55.1)	39	61.9 (48.8–73.9)	
≥200	11	73.3 (44.9–92.2)	24	38.1 (26.1–51.2)	
**History of opportunistic infections**					**0.578**
No	9	60.0 (32.3–83.7)	35	50.7 (38.4–63.0)	
Yes	6	40.0 (16.3–67.7)	34	49.3 (37.0–61.6)	
**Positive culture or Xpert**					**0.288**
No	26	26.0 (17.7–35.7)	32	20.3 (14.3–27.4)	
Yes	74	74.0 (64.3–82.3)	126	79.7 (72.6–85.7)	
**Clinical forms**					**<0.001**
Pleuropulmonary	74	74.0 (64.3–82.3)	114	72.1 (64.5–79.0)	
Extrapulmonary	25	25.0 (16.9–34.7)	20	12.7 (7.9–18.9)	
Disseminated	1	1.0 (0.0–5.4)	24	15.2 (10.0–21.8)	
**Clinical form (disease site)**					**<0.001**
Ganglionic	24	24.0 (16.0–36.6)	12	7.6 (4.0–12.9)	
Laryngeal, pulmonary and pleural	75	75.0 (65.3–83.1)	117	74.0 (66.5–80.7)	
Disseminated and meningeal	1	1.0 (0.0–5.4)	29	18.4 (12.7–25.3)	
**Acid-fast bacilli smear diagnosis**					**0.005**
Negative	51	56.7 (45.8–67.1)	56	37.6 (29.8–45.9)	
Positive	39	43.3 (32.9–54.2)	93	62.4 (54.1–70.2)	
**Platelet count, cells/mm3**					**0.008**
≤450,000	87	87.0 (78.8–92.9)	115	72.8 (65.1–79.6)	
>450,000	13	13.0 (7.1–21.2)	43	27.2 (20.4–34.9)	
**Morphological classification of red blood cell (based on size)**					**0.001**
Microcytosis	13	13.0 (7.1–21.2)	50	31.6 (24.5–39.5)	
Normocytosis	87	87.0 (78.8–92.9)	108	68.4 (60.5–75.5)	
**Morphological classification of red blood cells (based on Hb content)**					**<0.001**
Hypochromia	10	10.0 (4.9–17.6)	56	35.4 (28.0–43.4)	
Normochromia	90	90.0 (82.4–95.1)	102	64.6 (56.6–72.0)	

A: p-value: Chi-square or Fisher's exact test.

B: expressed values in Brazilian minimum wage: 1 MW = $270 (average values of the 4 years of the study [2015–2018]).

C: Drug use: includes the use of cannabis, cocaine, crack, hashish, ecstasy and solvents.

D: Comorbidities include one or more of the following: systemic arterial hypertension, diabetes mellitus, digestive disease, degenerative disease, hepatitis, kidney disease, neurological disease, chronic obstructive pulmonary disease or neoplasia/cancer.

The analysis of laboratory results showed that patients with anemia had higher values of C-reactive protein, leukocytes, neutrophils and platelets. In contrast, albumin, lymphocytes, MCV, MCH and CD4 cell values were lower in these patients (Table 2).

**Table 2:** Laboratory results at the time TB therapy initiation in patients with and without anemia.

	No anemia		Anemia		p-value1
	n	Median (IQR)	n	Median (IQR)	
**Albumin, g/dL (n = 209)**	85	3.7 (3.3–4.0)	124	2.8 (2.3–3.3)	<0.001
**C-reactive protein, mg/dL (n = 164)**	61	1.4 (0.4–3.7)	103	7.9 (4.7–11.9)	<0.001
**Leukocytes, cells/mm**^**3**^ **(n = 258)**	100	7115 (5885–8732)	158	7980 (6112–10387)	0.035
**Neutrophils, cells/mm**^**3**^ **(n = 258)**	100	4445 (3360–5986)	158	5484 (3845–7360)	0.006
**Lymphocytes, cells/mm**^**3**^ **(n = 258)**	100	1698 (1372–1999)	158	1394 (1024–1895)	0.001
**Eosinophils, cells/mm**^**3**^ **(n = 258)**	100	117.5 (63.7–222.5)	158	86.0 (0.0–240.5)	0.087
**Monocytes, cells/mm**^**3**^ **(n = 258)**	100	546 (397–743)	158	523 (327–766)	0.345
**Platelets, cells/mm**^**3**^ **(n = 258)**	100	278,500 (231,000–361,250)	158	361,500 (281,500–500,750)	<0.001
**MCV (fL) (n = 258)**	100	86.05 (82.47–88.72)	158	83.65 (78.57–87.80)	0.002
**MCH (pg) (n = 258)**	100	28.40 (27.15–30.12)	158	27.25 (25.20–28.70)	<0.001
**CD4 cell count, cells/μL (n = 78)**	15	304 (205–430)	63	145 (54–271)	0.003
**Viral load, copies/μL (n = 71)**	10	12,785 (413–77,808)	61	69,751 (2,295–162,035)	0.175

1: p-value: Mann-Whitney.

Normocytic normochromic anemia was the most common. Among the 158 patients with anemia, 60.8% (95% CI = 52.7–68.4) had normochromic normocytic anemia, 27.8% (95% CI = 21.0–35.5) hypochromic microcytic anemia and 11.4% (95% CI = 6.9–17.4) hypochromic normocytic anemia or normochromic microcytic anemia. Macrocytic anemia was not observed in any of the participants.

### Assessment of factors associated with anemia

The binary logistic regression to model the anemia outcome excluded 30 patients with meningeal and disseminated forms of TB due to low 2x2 table cell counts (<5) (Table 1). In the single-covariate models, we observed that anemia was more frequent in nonwhite patients and patients with fewer years of study, history of past alcohol use, drug use, low BMI, lower Karnofsky score, history of weight loss, previous TB treatment, hospitalizations, HIV infection (more frequent in those with CD4<200 cells/μL and interrupted ART), disseminated or meningeal clinical forms, positive smear diagnosis, higher platelet count, microcytosis or hypochromia. However, the multicovariate model (n = 228), adjusted for 12 variables, only showed 5 factors associated with anemia: history of weight loss >10%, hospitalizations, HIV infection, microcytosis and high platelet count (Table 3).

**Table 3:** Single and multicovariate logistic regression models of factors associated with anemia at the time of initiation of TB therapy (n = 228).

	Crude OR (95%CI)	p-value	Adjusted OR* (95% CI)	p-value
**Race**				
White	1		1	
Nonwhite	2.23 (1.34–3.72)	0.002	1.87 (0.93–3.71)	0.075
**Schooling**				
None/incomplete elementary	2.79 (1.45–5.37)		1.92 (0.80–4.62)	
Complete elementary	2.20 (1.21–3.98)		1.08 (0.49–2.38)	
High school/university	1	0.003	1	0.315
**Alcohol**				
Never	1		1	
Quit	2.67 (1.42–5.00)		1.88 (0.80–4.44)	
Current	1.18 (0.63–2.22)	0.005	1.11 (0.45–2.74)	0.271
**Drug use**^**A**^				
No	1		1	
Yes, noninjecting	2.14 (1.13–4.04)	0.020	0.94 (0.37–2.36)	0.894
**Karnofsky score**				
≤80	3.18 (1.88–5.37)		1.51 (0.75–3.06)	
90 or 100	1	<0.001	1	0.247
**Weight loss history (>10%)**				
No	1		1	
Yes	5.52 (3.03–10.05)	<0.001	2.35 (1.11–4.97)	0.025
**Previous TB**				
No	1		1	
Yes	1.94 (1.04–3.63)	0.038	1.61 (0.71–3.65)	0.253
**Hospitalization**				
No	1		1	
Yes	6.15 (2.88–13.12)	<0.001	2.91 (1.16–7.29)	0.022
**HIV infection status**				
No	1		1	
Yes	4.39 (2.33–8.27)	<0.001	4.04 (1.70–9.57)	0.002
**Acid-fast bacilli smear diagnosis**				
Negative	1		1	
Positive	2.17 (1.27–3.70)	0.004	1.92 (0.94–3.92)	0.072
**Platelets count, cells/mm3**				
≤450,000	1		1	
>450,000	2.50 (1.27–4.94)	0.008	2.53 (1.07–5.98)	0.035
**Morphological classification of red blood cell (based in size)**				
Microcytosis	4.00 (1.58–6.07)		2.36 (1.02–5.44)	
Normocytosis	1	0.001	1	0.044

A: Drug use: includes the use of cannabis, cocaine, crack, hashish, ecstasy and solvents; OR = odds ratio, CI = confidence interval.

*Adjusted OR: Hosmer and Lemeshow Test (p = 0.691).

## Discussion

The main results of this study were as follows: a) anemia was very common, occurring in 61.2% of TB cases; b) there was a predominance of mild and moderate anemia, and severe anemia was uncommon; c) normocytic normochromic anemia was the most common, followed by hypochromic microcytic anemia, and macrocytic anemia did not occur in any patient; d) anemia was associated with factors indicating the severity of TB.

We found a prevalence of anemia similar to those observed in studies carried out in Asia [7] and Africa [5, 11, 13, 15, 23]. However, Lee et al. showed a lower prevalence of anemia in South Korea (31.9%). This may be because they excluded participants with medical conditions that could cause anemia, such as bleeding, nutritional deficiency, malignancy, HIV infection, comorbidities and medication use [24], making their population different from ours.

Despite the high prevalence of anemia in our study, most cases were mild or moderate, and severe anemia was uncommon. The prevalence of anemia we found was lower than that in three other studies, namely, two performed in Brazilian hospitalized patients [17, 18] and another in TB-HIV patients [25], which could explain the higher prevalence of anemia observed by these authors. We found that anemia was associated with HIV infection, hospitalization and a history of weight loss >10%, showing that more severe TB patients were anemic. We also observed that almost all patients in the disseminated + meningeal TB group had anemia (29 out of 30 cases).

All of these findings may corroborate the idea that anemia is more prevalent in more severe forms of TB. HIV infection has a substantial impact on TB-associated mortality and is higher in cases of TB-HIV without ART use, as was already shown in a study from our group [26]. The disseminated and meningeal TB clinical forms have been shown to have higher mortality rates [27–30]. Poor health status in TB patients is a common cause of hospitalization [31], and a history of weight loss, which is a sign of malnutrition and greater disease severity, is considered a predictor of TB death [10, 32].

We observed that conditions predicting nutritional deficiency (such as low BMI, history of weight loss, and low albumin levels) were more frequent in anemic than in nonanemic patients, and C-reactive protein, a marker of an active inflammatory process, was significantly higher in patients with anemia. Other studies have also shown an association of anemia with hypoalbuminemia [17, 23], increased C-reactive protein [7, 17, 23] and decreased BMI [8, 15, 16]. These observations, together with the evidence of decreased MCH and MCV in patients with anemia and the significant prevalence of microcytic hypochromic anemia (27.8% of anemia cases), allow us to speculate that important causes of anemia in TB patients may be iron nutritional deficiency and/or its decreased availability to erythropoiesis due to alterations of its metabolism in inflammation. The higher platelet counts in patients with anemia may corroborate this argument because lower iron availability in the bone marrow leads to the production of megakaryocytes with higher ploidy, which may release more platelets than lower-ploidy ones [33].One study that assessed the causes of anemia estimated that 15.0% to 75.9% of cases were inflammation anemia and 0.0% to 58.0% were iron-deficiency anemia [4]. This variation may be due to different criteria and study designs.

Macrocytic anemia was not observed among the patients in our study. The lack of zidovudine among the antiretroviral drug regimens could have contributed to this finding [34], but zidovudine is not currently recommended by Brazilian guidelines.

The study had limitations. 1) Because it was retrospective, some data, such as on iron, ferritin, hepcidin and transferrin, were not available to help us better assess the etiology of the anemia. 2) For the same reason, other data, such as BMI, income, albumin and C-reactive protein, had more than 10% missing results and could not be included in the multicovariate binary logistic regression analysis.3) Some ORs were high and had wide confidence intervals due to small counts in some covariates. 4) We did not have any information about the following: helminth, protozoan, viral or bacterial infections; chronic bleeding; low food intake; hereditary anemia; or drugs with the potential to cause anemia. Thus, we could not assess the impact of these conditions on the study results.

In conclusion, anemia was highly prevalent among TB patients. There was a predominance of normochromic normocytic anemia, but hypochromic microcytic anemia was also common. Anemia was associated with clinical conditions, such as HIV infection, significant weight loss and hospitalization. Anemia was also highly prevalent in patients with the most severe clinical forms of TB, suggesting that anemia could be used as a biomarker of TB severity. As complete blood count is a simple and available test worldwide, it would be very useful for identifying severe cases of TB in clinical practice.

## Supporting information

S1 Data (SAV)

S2 Data (XLSX)
