# Correction: Anemia in tuberculosis cases: A biomarker of severity?

**DOI:** 10.1371/journal.pone.0249545

**Published:** 2021-03-29

**Authors:** Edson Beyker de Mendonça, Carolina AranaStanis Schmaltz, Flavia Marinho Sant’Anna, Alexandre Gomes Vizzoni, Daniela Palheiro Mendes-de-Almeida, Raquel de Vasconcellos Carvalhaes de Oliveira, Valeria Cavalcanti Rolla

The Funding statement is incorrect. The correct Funding statement is: “This study was financed in part by the Coordination for Improvement of Higher Education Personnel (CAPES)—Finance Code 001”
